# The positive impact on translational research of Fondazione italiana di ricerca per la Sclerosi Laterale Amiotrofica (AriSLA), a non-profit foundation focused on amyotrophic lateral sclerosis. Convergence of ex-ante evaluation and ex-post outcomes when goals are set upfront

**DOI:** 10.3389/frma.2023.1067981

**Published:** 2023-08-04

**Authors:** Stefania Guareschi, Maddalena Ravasi, Danila Baldessari, Silvia Pozzi, Tiziana Zaffino, Mario Melazzini, Anna Ambrosini

**Affiliations:** ^1^Fondazione AriSLA ETS, Milan, Italy; ^2^Fondazione Telethon ETS, Milan, Italy

**Keywords:** amyotrophic lateral sclerosis, ALS research, relative citation ratio, RCR, research assessment

## Abstract

Charities investing on rare disease research greatly contribute to generate ground-breaking knowledge with the clear goal of finding a cure for their condition of interest. Although the amount of their investments may be relatively small compared to major funders, the advocacy groups' clear mission promotes innovative research and aggregates highly motivated and mission-oriented scientists. Here, we illustrate the case of Fondazione italiana di ricerca per la Sclerosi Laterale Amiotrofica (AriSLA), the main Italian funding agency entirely dedicated to amyotrophic lateral sclerosis research. An international benchmark analysis of publications derived from AriSLA-funded projects indicated that their mean relative citation ratio values (iCite dashboard, National Institutes of Health, U.S.) were very high, suggesting a strong influence on the referring international scientific community. An interesting trend of research toward translation based on the “triangle of biomedicine” and paper citations (iCite) was also observed. Qualitative analysis on researchers' accomplishments was convergent with the bibliometric data, indicating a high level of performance of several working groups, lines of research that speak of progression toward clinical translation, and one study that has progressed from the investigation of cellular mechanisms to a Phase 2 international clinical trial. The key elements of the success of the AriSLA investment lie in: (i) the clear definition of the objectives (research with potential impact on patients, no matter how far), (ii) a rigorous peer-review process entrusted to an international panel of experts, (iii) diversification of the portfolio with *ad hoc* selection criteria, which also contributed to bringing new experts and younger scientists to the field, and (iv) a close interaction of AriSLA stakeholders with scientists, who developed a strong sense of belonging. Periodic review of the portfolio of investments is a vital practice for funding agencies. Sharing information between funding agencies about their own policies and research assessment methods and outcomes help guide the international debate on funding strategies and research directions to be undertaken, particularly in the field of rare diseases, where synergy is a relevant enabling factor.

## Introduction

The field of rare diseases offers numerous virtuous examples of charities promoting cutting-edge research. Some, such as the Rare Disease Foundation (Rare Disease Foundation^1^), largely invest in seed funding research and development; others, like Fondazione Telethon (Aiuti et al., 2022), promote research along all the pipeline with the vision to make the cure available to patients. Most charities are disease-specific, such as the Juvenile Diabetes Research Foundation (JDRF—Type 1 Diabetes Research Funding and Advocacy^2^), the Cystic Fibrosis Foundation (Home | Cystic Fibrosis Foundation (cff.org)^3^, or the Parent Project Muscular Dystrophy [Parent Project Muscular Dystrophy (PPMD) | Fighting to End Duchenne (parentprojectmd.org)^4^], just to mention a few investing in frontier research to promote development of new therapies for their diseases. Here, we illustrate the experience of Fondazione italiana di ricerca per la Sclerosi Laterale Amiotrofica (AriSLA) to discuss how even a small funding agency can make a difference and contribute high-quality scientific knowledge to progress toward new therapeutic developments. AriSLA is a research funding agency started in 2008 by the will of the Italian amyotrophic lateral sclerosis (ALS) patient organization Associazione Italiana Sclerosi Laterale Amiotrofica Onlus, in partnership with Fondazione Cariplo (a philanthropic bank foundation), Fondazione Telethon (the main Italian funding agency for genetic diseases), and Fondazione Vialli e Mauro per la Ricerca e lo Sport Onlus (a philanthropic non-profit organization) to address the urgency of ALS patients to find a cure for their disease.

ALS is a severe progressive neurodegenerative disease of adulthood, caused by the loss of spinal, bulbar, and cortical motor neurons, which leads to paralysis of the voluntary and respiratory muscles (Masrori and Van Damme, 2020). Typically, it affects people aged between 40 and 70, with an estimated annual incidence of 1–3 cases per 100,000 persons (Masrori and Van Damme, 2020; Vasta et al., 2021). Life expectancy after diagnosis is 3–5 years on average, but the clinical course of the disease is rather heterogeneous, as is the pathophysiology, which is believed to depend upon complex interactions between genetic, environmental, and lifestyle factors (Kiernan et al., 2021). Several complex pathophysiological mechanisms are involved in ASL at the cellular level, making investigation very difficult (Van Damme et al., 2017; Kiernan et al., 2021). A family history is reported in about 10% of patients, and several genes associated with ALS have been identified. The remaining 90% of ALS cases is considered sporadic, but the possibility that genetic variations influence the individual susceptibility to ALS was suggested (Masrori and Van Damme, 2020). Although effective therapeutics are not currently available to patients, the scenario is rapidly changing and several innovative therapeutic strategies are currently being investigated, particularly for specific genetic forms (Garam et al., 2020; Miller et al., 2022). In 2022, the National Institute of Neurological Disorders and Stroke (NINDS) of the National Institutes of Health U.S. (NIH) set a Steering Committee that help form and coordinate 5 working groups to examine the ALS research landscape and draft recommendations for a Strategic Plan, based on the challenges and key priorities identified.^5^ Each working group, which included different stakeholder representatives, highlighted 3 top research priorities to be taken into consideration to draw the future directions of ALS research. A Draft document was open to Public Comments in the period November-December 2022, and the finalized document was made available online.^6^

After more than 10 years of investment in research, recently AriSLA carried out an in-depth assessment to provide its Board of Directors with a complete picture of the quality and directions of its research, for an informed decision-making on its future strategies. The Web of Science Core Collection^TM^ by Clarivate^TM^ ^7^ provided a list of peer-reviewed publications acknowledging AriSLA funds, as well as their level of influence by the scientific community in the Web of Science Citation Report. In addition, a bibliometric international benchmark analysis was conducted using the relative citation ratio (RCR) metric provided by the iCite platform of the NIH Office of Portfolio Analysis (Santangelo, 2017; iCite | New Analysis | NIH Office of Portfolio Analysis^8^) This tool is adopted by the NIH and other main research institutes, particularly in the United States for the analysis of their investment (Santangelo, 2017; Surkis and Spore, 2018; Rechtman et al., 2022), or by learned societies for comparison of research productivity amongst academic faculty (Reddy et al., 2020; Dijanic et al., 2022; Patel and Ali, 2022). However, its use by funding agencies and charities for international benchmark is still rather limited, or at least this information is not easily accessible. Results derived from the analysis performed by AriSLA that are herein reported strongly support its helpfulness also in the case of a small non-profit organization.

The bibliometric results were integrated with a qualitative analysis of the AriSLA funded projects' performance. Overall, this assessment provided an interesting picture of the AriSLA portfolio. Despite the publication outputs were relatively small due to the limited availability of funds and number of projects, the scientific results derived from AriSLA grants were well received by the publishers and had strong impact on the dissemination of knowledge by the international scientific community. The results of the analysis also suggest that a rigorous process of allocation of funds, based on competition, transparency, and careful scientific evaluation, contributed to fulfilling AriSLA's mission and to building knowledge on ALS that has already been highly influential at international level.

## Methods

### Research project selection and management process

AriSLA grants are regularly awarded on a competitive basis, according to the selection criteria indicated in the call for research projects. The relevance of the proposed research to ALS is a prerequisite to admitting applications. The peer review process is conducted by the AriSLA International Scientific Committee, a panel of experts in the field, not working in Italian centers to minimize the risk of conflicts of interest and consists of two consecutive phases: a preliminary triage followed by full proposal evaluation of the selected applications. The AriSLA call for research projects is issued annually and admits two types of applications: full grants (FG) and pilot grants (PG). Reviewers evaluate each research proposal based on scientific quality, innovation, feasibility, and potential impact on ALS, but a different emphasis is placed on criteria for FG and PG. The AriSLA scientific office periodically monitors the outputs of the funded projects, through scientific reports, site visits, surveys to principal investigators (PIs), and scientific events.

### Bibliometric analysis

Publications acknowledging the contribution of AriSLA grants were indexed on Web of Science Core Collection^TM^ by Clarivate^TM^. This bibliometric tool was found to be more useful than PubMed National Library of Medicine (https://pubmed.ncbi.nlm.nih.gov/) because it allows searching for articles based on acknowledgment to funders (last access July 3, 2023). Moreover, queries performed on the Web of Science's “Citation Report”' module provided information on the “Sums of the Time Cited” (total citations) and “Average Citations per Item” (mean citations per article) (last access July 3, 2023). A few additional publications in Journals lacking the Acknowledgment section, and therefore not retrieved by the Web of Science search, were known to AriSLA having been reported directly by the PIs in their final scientific reports and their PubMed IDs were added to the list for further bibliometric analysis.

The PubMed IDs of AriSLA-derived publications (373 publications) were used for bibliometric analysis based on the RCR, an article-level metric developed by the NIH Office of Portfolio Analysis (Hutchins et al., 2016) that is calculated as the number of cites per year of each paper, divided by the average citations per year received by NIH-funded papers in the same field. Fields are sampled for each article by using its co-citation network. Articles under considerations are therefore benchmarked to publications derived from NIH-R01 funded projects for the referring year, where RCR value equal to 1.0 corresponds to the median value of NIH-R01 derived publications, separating 50% of articles with a higher citation rate (values above 1.0) from the 50% with a lower citation rate (values below 1.0). The RCR values are provided through the iCite dashboard, which is freely accessible online (iCite | New Analysis | NIH Office of Portfolio Analysis) (data collection regarding AriSLA publications: March 28, 2023).

The iCite tool retrieved all 373 AriSLA publications, however the RCR analysis was performed only on the 325 papers published in the period 2010–2021, the other 48 (2022–2023) being too recent to provide RCR values or with provisional RCR values, not reliable enough, according to the developers of the iCite platform, for accurate analysis.^9^

An in-depth analysis was conducted on the original articles only. These were grouped into 6 thematic categories based on the keywords specified by the PIs within the related proposals and verified by the AriSLA scientific office, 5 of which representing some of the main preclinical research areas of interest in ALS (Van Damme et al., 2017) and one category defined “clinical research,” including both clinical and technological projects. The 5 categories of projects and related articles addressing preclinical research were grouped as follows: (i) “autophagy and stress response,” including topics on protein misfolding and aggregation, stress granules, post-translational modifications, DNA Damage, stress response; (ii) “excitation and energy metabolism,” including topics on involvement of mitochondria and endothelial reticulum, hypermetabolism and hyperexcitability, calcium signaling, excitatory and inhibitory signaling; (iii) “genetics and genomics,” including topics on new mutation discovery, target identification, gene function; (iv) “non-cell autonomous mechanisms,” including topics on oligodendrocytes regeneration, microglia and immune system functions and modulation, endothelial cells role, trophic factors, exosomes, neuromuscular junction, adrenergic modulation; and (v) “RNA metabolism and epigenetics,” including topics on RNA expression, modulation, regulation, maturation and editing, DNA methylation and acetylation, RNA-targeted therapeutics. Original articles were also analyzed using the “Triangle of Biomedicine” (ToB), a machine learning-based iCite tool that shows the relative density of articles in different areas of a triangle, whose corners represent Molecular/Cellular biology (Mol/Cell), Animal, and Human -oriented research (Hutchins et al., 2019a,b). The position of a publication within the triangle is based on its Medical Subject Headings (MeSH) ontology (Weber, 2013; Hutchins et al., 2019b). Total citations and average citations per article were also collected via the iCite dashboard Citation Module (last access July 3, 2023).

Key publications acknowledging the funded projects were mentioned in the Results and Discussion sections. The publications' choice was based on one or more of the following criteria: (i) relevance for the topic discussed; (ii) pivotal paper or most recent publication by the author on the topic; (iii) RCR ≥1 (if available). This list of articles is proposed for illustrative purposes only and is not intended to be exhaustive of all relevant publications derived from AriSLA-funded projects.

### Survey among grantees

A survey among 80 PIs (PIs, project's coordinators or partners) recipient of the 61 AriSLA grants completed by December 2019 was conducted in February-May 2020 (online Microsoft forms). The survey was filled in by 51 PIs providing information on 46 awarded projects (response rate: 64% of PIs, corresponding to 75% of projects). The questionnaire included 38 questions grouped into 6 domains: (1) PI's personal information; (2) information on the AriSLA award(s); (3) PI's expertise on ALS; (4) grant follow up; (5) satisfaction on AriSLA management; (6) participation in AriSLA scientific events and suggestions. This manuscript includes aggregated information collected in domains 2 to 4, which investigated the PI's expertise on ALS at the time the project was funded, the impact of the AriSLA award on the interaction of the research teams with national/international ALS networks and on the obtainment of additional funds to further develop their ALS research.

## Results

### AriSLA research portfolio

Since 2009, AriSLA has launched 15 Calls for projects (average success rate of 7 ± 1%), dedicated to FG or PG proposals. Essential requisite for both types of proposals to be fundable was that science content had to be of high value, original and ground-breaking with respect to current knowledge. The two programs, however, placed different emphasis on the goals they intended to achieve, with FGs supporting studies progressing from solid and established lines of research, and PGs aimed to open new innovative fields and explore riskier ideas. AriSLA has so far financed 98 projects, 52 FGs and 46 PGs, supporting 143 Italian research teams, with an overall investment of 14.98 million euros. The majority of AriSLA investment has been awarded to basic (64 projects) and preclinical (20 projects) research. These studies covered many of the cutting-edge research topics in the ALS field (Van Damme et al., 2017), namely: autophagy and stress response, excitation and energy metabolism, genetics and genomics, non-cell autonomous mechanisms, and RNA metabolism and epigenetics. AriSLA also supported 4 interventional Phase 2 clinical trials on small molecule repurposing and 6 observational clinical studies on diagnosis or predictive and prognostic biomarkers, as well as 4 projects aimed at developing new assistive technology tools to improve patients' quality of life. Fund allocation and project results have been regularly monitored to ensure their alignment with AriSLA mission and for transparent communication to the public. Since 2009, a scientific Advisory Board has supported the president and the scientific office in strategic decisions relating to AriSLA funding initiatives.

### Bibliometric analysis

AriSLA does not apply journal-based metrics, such as the Journal Impact Factor (JIF) or the Hirsch-index, to assess candidates to its calls or the productivity of its grantees. Regarding the JIF, several analyses have highlighted the main shortcomings of this metric when it is adopted as a proxy to evaluate the productivity and influence of scientists through their research publications (e.g., 2003) and such considerations led a group of editors and publishers to issue the 2012 San Francisco Declaration on Research Assessment - DORA declaration^10^ (Cagan, 2013). As for the Hirsch-index (Hirsch, 2005), it is used to evaluate the research outputs of an individual researcher and compare its activity with peers by measuring the number of articles published and the citations received. This index, however, is strongly dependent on the research field and seniority of a researcher; therefore, it is not appropriate to evaluate scientists with different career length. The selection process of grant applications adopted by AriSLA considers the performance of the PI as part of the feasibility of the proposal. However, this assessment is not based on publication metrics and includes the expert opinion of the reviewers on the quality of previous results, also considering the level of seniority of the PI.

Overall, 373 PubMed-indexed publications associated with 78 AriSLA grant projects were identified, of which 281 original articles and 92 reviews. The average number of publications per project was 4.78, with a range of 1–17 for original articles and 1–9 for reviews. As expected, FG projects were more productive than PG projects, with averages of 6.56 and 2.17 publications per project, respectively.

For the portfolio analysis herein presented, a bibliometric evaluation was carried our using the iCite platform [iCite | New Analysis | NIH Office of Portfolio Analysis (see text footnote 8)] of the NIH Office of Portfolio Analysis (Hutchins et al., 2016; Santangelo, 2017). In addition, citations of publications derived from AriSLA funded projects obtained through the iCite dashboard were compared to values provided by the Web of Science Citation Report by Clarivate^TM^ (www.webofscience.com).

The iCite dashboard (iCite | New Analysis | NIH Office of Portfolio Analysis^8^) offers 3 types of analysis; influence (Hutchins et al., 2016), translation (Weber, 2013; Hutchins et al., 2019b), and citations (Hutchins et al., 2019a), which were applied to AriSLA publications.

#### Influence module

This module is based on the RCR metric, which was developed by the NIH Office of Portfolio Analysis to overcome the flaws of the JIF. RCR is a validated article-level measure, field and time—normalized, that allows comparison of an even small collection of articles by benchmarking the citation rates of NIH-R01 derived article in the respective area of research (Santangelo, 2017). As indicated by the developers, it should not be considered as a direct index of impact or quality of research or be used to assess individual researchers for making funding decisions, as these evaluations require more comprehensive analyses, including qualitative human judgement. Instead, it can be considered an index of the influence of the outputs of groups of researchers or even that of a single article in a certain field (Santangelo, 2017). This approach has been applied to AriSLA-derived publications and is reported as a measure of their influence in the international peer network. Since RCR value equal to 1.0 corresponds to the median value of NIH-R01 publications, values above 1.0 are indicative of articles in the NIH higher 50% citation rate, while values below 1.0 indicate those falling in the NIH 50% lowest citation rate. Highlight has been put on groups of articles with mean RCR values equal or higher than 2.0, which correspond to the top 25% citation rate of NIH-derived publications.

The RCR analysis concerned the 325 publications of the period 2010–2021, which derived from 70 AriSLA projects (41 FG and 29 PG projects). The original articles were 248 (mean RCR = 2.17 ± 0.19; median = 1.49), while reviews were 77 (mean RCR = 3.45 ± 0.96; median = 1.86). The FG projects generated 263 publications (196 original articles and 67 reviews), of which 178 with RCR ≥1.0 (68%), and 98 with RCR ≥2.0 (37% of total FG publications). Interestingly, PG projects were also highly productive, generating 62 publications (52 original articles and 10 reviews), of which 40 with RCR ≥1.0 (65%), and 25 with RCR ≥2.0 (40% of total PG publications).

**Figure 1:**
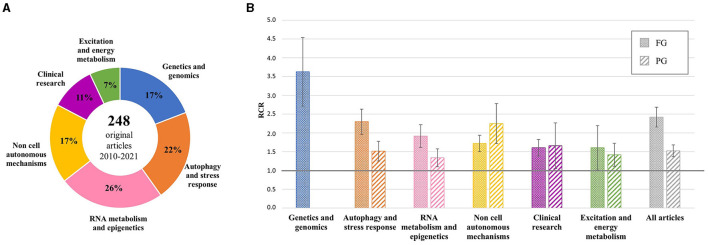
Distribution of original articles by topic and corresponding RCR. **(A)** AriSLA-derived original articles grouped by scientific topic of the referring proposal; the percentage with respect to the total number of original articles is reported for each group. **(B)** Mean RCR values (±standard error of mean) of original articles by topic compared to the NIH value of reference (RCR = 1.0). Columns with dots concern full grant (FG)-derived articles; columns with stripes concern pilot grant (PG)-derived articles. RCR, relative citation ratio. Publication period 2010–2021.

Additional analysis was performed on the 248 original articles grouped by scientific topic (Figure 1A). The mean RCR values were >1.0 in all groups, both for FG and PG projects, and >2.0 for FG articles generated by genetic studies and research on autophagy and stress response (top 25% of NIH benchmark) and for PG articles on non-cell autonomous mechanisms, indicating a very high influence of these articles by the ALS community (Figure 1B).

**Figure 2:**
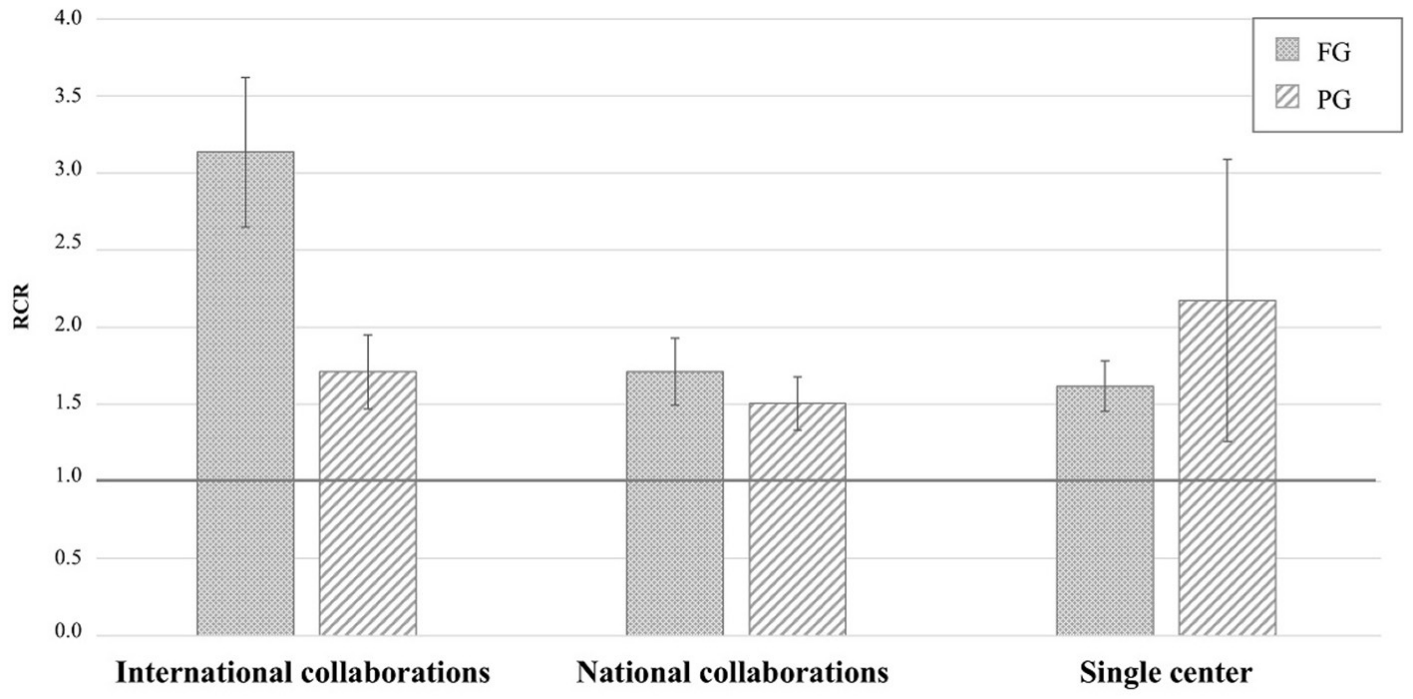
Distribution of original articles by type of Author's collaboration and corresponding RCR. Mean RCR values (±standard error of mean) of AriSLA-derived original articles compared to the NIH reference value (RCR = 1.0). The type of collaboration reported is based on the list of authors included in the publications that acknowledged, among others, AriSLA funding. Columns with dots concern full grant (FG)-derived articles; columns with stripes concern pilot grant (PG)-derived articles. RCR, relative citation ratio. Publication period 2010–2021.

The highest RCR values derived from collaborative international collaborations, such as the genetic consortia that led to ALS gene discovery (e.g., Majounie et al., 2012; Wu et al., 2012; Johnson et al., 2014; Nicolas et al., 2018), and the working groups on RNA metabolism (e.g., Ayala et al., 2011) or autophagy and cellular stress response (e.g., Ganassi et al., 2016), followed by those derived from national collaborations and individual research groups (Figure 2). The iCite dashboard does not report RCR values based on the investigator's position in the author list. AriSLA staff then reviewed the 138 original articles derived from collaborative projects with RCR values ≥1.0, to define AriSLA grantee's contribution to the study, determining that they were first, last, or corresponding author in 45 out of 74 papers derived from international collaborations and in 53 out of 64 related to national groups. These data indicate that the AriSLA support has helped strengthen scientific interactions and leadership of its grantees both nationally and internationally.

The bibliometric results of PGs were rather unexpected, considering that PG projects are innovative and exploratory in nature, the purpose of which is to generate those preliminary data that help build sufficient knowledge for more robust research developments. Therefore, their performance should be highlighted, albeit lower than that of the FG projects. Indeed, not only did a large number of PG projects (29 of total 39 projects started before 2021) selected on the above criteria provide peer-reviewed publications, but also mean RCR values of original articles were above 1.0 in all research categories addressed by these studies (although for some areas the total number of PGs and related publications was rather limited) (Figure 1B). Furthermore, several PIs managed to involve international and national collaborators in their projects (Figure 2), thereby broadening their research network. Overall, these bibliometric results support the view that the highly competitive grant selection process helped identify promising studies and that the PIs made considerable effort to achieve internationally recognized results, a result which is valid for both grant categories.

#### Translation module and the triangle of biomedicine

The iCite Translation Module is meant to offer a quantitative way to gain information on the translational potential of the published results. It is based on a computational model that combines several features to predict to which degree a paper will be of translational/clinical utility. A visual tool, the ToB, plots each article into a graph according to its MESH terms, which indicate its closeness to molecular, animal or human -focused research, together with other terms related to disease, chemical/drug and diagnosis/therapeutic approaches. The iCite dashboard also reports those papers already cited by clinical articles as additional component of their translational potential. The ToB, together with the citation history concur to generate the Approximate Potential to Translate (APT) score, an estimate of the likelihood of an article to be cited in future clinical articles that can be differently influenced by one or the other parameters depending on the nature of the paper itself (Hutchins et al., 2019b).

**Figure 3:**
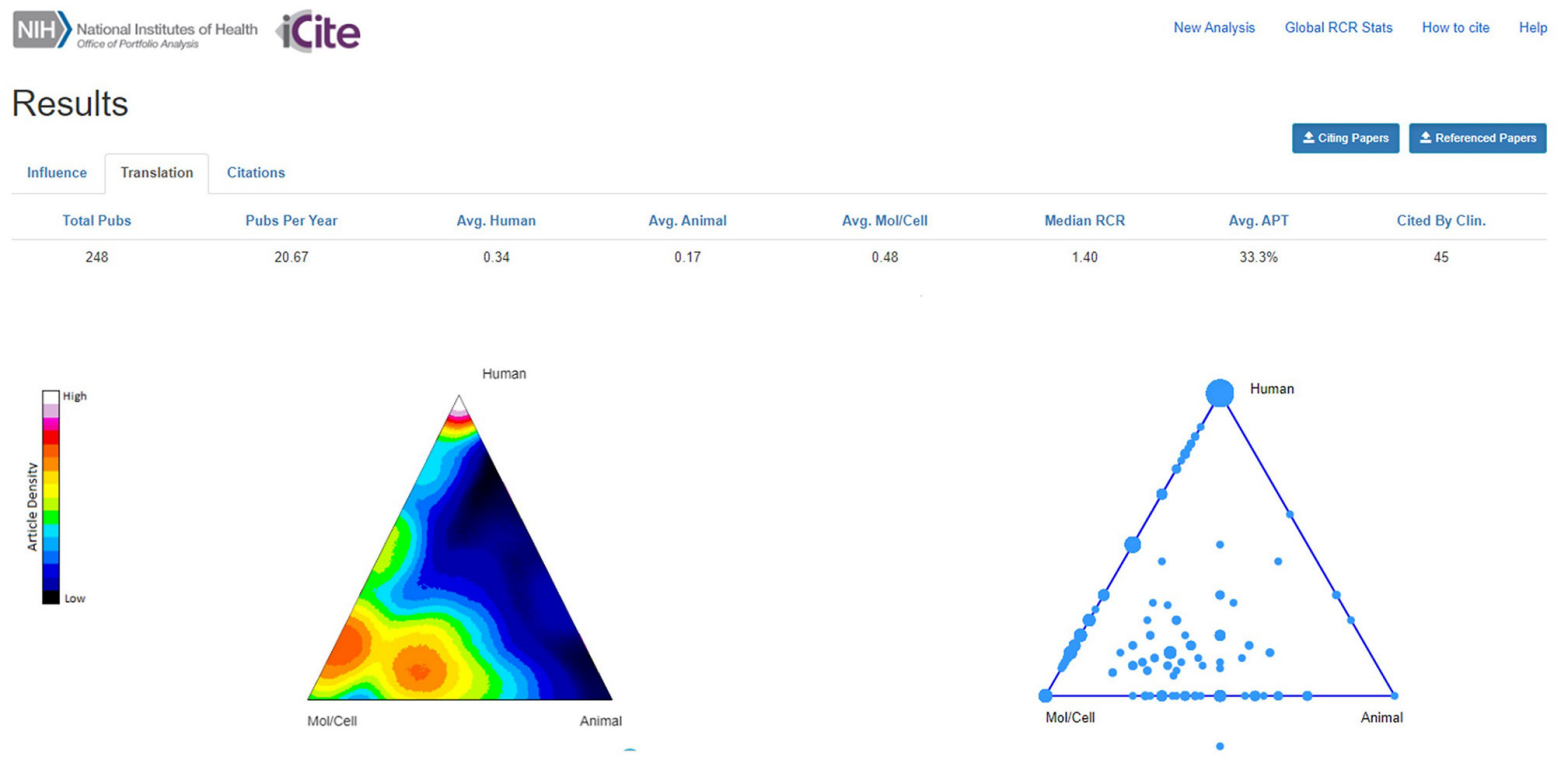
Density plot of original articles in the triangle of biomedicine. The density plot shows relative density of articles in an area of the triangle as pseudocolor, with white and “hotter” colors representing the higher article density, while the bubble plot indicates where papers are located. Triangle corners represent Animal (bottom right vertex), Molecular/Cellular biology (Mol/Cell; bottom left vertex), and Human (top vertex) -oriented research. Multiple papers can have the same coordinates; the size of the bubble is proportional to the number of articles. The dot outside the bubble plot represents an article that had no MeSH terms in any of the categories Human, Animal, or Mol/Cell biology. Avg., average; APT, Approximate Potential to Translate; Clin., Clinic. Publication period 2010-2021.

The 248 AriSLA-derived original articles were plotted in the ToB (Figure 3). The highest density of articles mapped in the bottom part of the ToB, under the Mol/Cell vertex, as expected given that 65% of the funded projects were on basic research. Articles derived from preclinical projects plotted in the hotspot above the Mol/Cell-Animal axis or in the center of the ToB, confirming their actual preclinical nature, in line with the indications of the developers of the algorithm for publications that map in the center of the ToB and next to the bisector of the Mol/Cell-Animal axis and the Human vertex (Weber, 2013; Hutchins et al., 2019b). It will be interesting to continue to monitor this trend, as only 11 out of 20 grant projects with preclinical focus started before 2021 and had already published results at the time of this publication. A high density of articles ranked below the Human vertex, being the outcomes of clinical or technological research, as well as of genetics and genomics studies. The last topic was also well represented along the Mol/Cell-Human edge, together with papers on RNA metabolism and epigenetics.

Forty-five AriSLA-derived original articles (18.1%) had already been cited by clinical papers (Figure 3; “Cited By Clin.”). This value was well in line with the estimates proposed by Hutchins and collaborators, who reported that only about 25% of the publications considered in their large analysis reached this target, being about 18% for research articles (Hutchins et al., 2016). About half (23) of the AriSLA-derived articles in this group were directly focused on human-oriented research, deriving from projects focused on genetics (12 articles) (e.g., Majounie et al., 2012; Wu et al., 2012), clinical studies (8 articles) (e.g., Riva et al., 2019), or technological devices (3 articles) (e.g., Aloise et al., 2012; Caligari et al., 2013). Their average RCR was 6.01 ± 1.49 (median = 2.71), with only two papers below 1. The other half (22 articles) were generated by basic or preclinical research projects, focused on autophagy and stress response (12 articles) (e.g., Ganassi et al., 2016; Cristofani et al., 2018), RNA metabolism and epigenetics (5 articles) (e.g., Budini et al., 2012; Errichelli et al., 2017; Gagliardi et al., 2018), non cell autonomous mechanisms (3 articles) (e.g., Pasetto et al., 2017; Garofalo et al., 2020), and excitation and energy metabolism (2 articles) (e.g., Belli et al., 2019). Their average RCR was 3.57 ± 0.72 (median = 2.56), with only 2 paper below 1. The average APT score was 33.3% for the 248 articles (Figure 3) and raised to 54.9% for the sub-group of original articles that had already been cited by clinic (not shown).

#### Citations module

This module is based on the NIH Open Citation Collection, a dataset generated by the NIH Office of Portfolio Analysis from unrestricted data sources such as MedLine, PubMed Central, and CrossRef, with the aim of making the citation statistics publicly available through the NIH *iCite* analytic platform (Hutchins et al., 2019a). The 248 articles received 12,120 citations, with a mean number of citations per publication of 48.87 ± 5.59 (median = 24). Of note, the citations of the 45 articles already quoted by clinical publications were 5,495 (45% of all citations), with a mean number of citations per article of 122.11 ± 25.43 (median = 56), indicating that these articles had a particular influence within the ALS scientific community. Citation values were also collected via the Web of Science Citation Report, which is based on the Core Collection^TM^ by Clarivate^TM^ database. According to the Web of Science Citation Report, the total citations' value of the 248 original articles was 12,045, with an average citation per article of 48.57, while for the selected 45 articles already cited by clinic total citations' value was 5,435, with an average citation per article of 120.78. The almost identical results obtained via iCite and Web of Science suggested a full overlap between the two datasets and confirmed the validity of both collections, with the advantage of iCite being a free tool available online.

#### Typology of journal

Results from the AriSLA projects have been published in a wide range of scientific journals, mainly in the fields of cell biology, human genetics, or neuroscience and neurodegeneration. The 10 most common (in descending order, with some journals tied, therefore all together occupying the first 7 positions) were: Scientific Reports, Neurobiology of Aging, International Journal of Molecular Sciences, Human Molecular Genetics, Neurobiology of Disease, Nature Communications, Cell Death & Disease, Journal of Biological Chemistry, Journal of Neurology, Nucleic Acids Research (92 out of 281 original articles, period 2010–2023; last access July 3, 2023; 5 or more articles/journal). Other seven journals ranked equally at the 8^th^ position (4 articles/journal), including Amyotrophic Lateral Sclerosis and Frontotemporal Degeneration, the leading journal for ALS, which, interestingly, was not at the top of the list, not even for clinical articles. Neurobiology of Aging was the first journal selected for FGs (17 papers), followed by International Journal of Molecular Sciences and Scientific Reports (each with 10 publications), while results derived from PGs were mainly published on Scientific Reports (11 articles), followed by Cell Death & Disease (4 articles), and International Journal of Molecular Sciences (3 articles). Scientific Reports was the top journal also for articles already quoted by clinical publications (5 articles), followed by Autophagy and Human Molecular Genetics and Neuron (3 articles/journal). This widespread typology of journals, and their broad focus, is not surprising when you consider that the vast majority of AriSLA projects concerned basic and preclinical research.

### Qualitative analysis of AriSLA-funded projects and PI performance

A survey was conducted in 2020 on PIs of completed single or multicenter projects. All 51 respondents were still working on ALS at the time of the survey; 21 had not worked on ALS before receiving the AriSLA grant and 14 of them started with the support of a PG; 10 PIs in the newcomers' group were under the age of 40 at the time of the award. Thirty respondents (59%) successfully raised additional public or private funding for their ALS research by including results from AriSLA-funded projects in the new application (respectively, 71% and 49% of FG and PG PIs), with 43 grants attracted from main ALS charities outside Italy e.g., ALS Association, United States (Dedicated to Finding a Cure for ALS | The ALS Association^11^), Fondation Thierry Latran, France^12^, and Motor Neuron Disease Association, UK (MND Association | Fighting motor neurone disease^13^), local philanthropic bank foundations or national (Italian Ministries of Health or Research) and international (European Union programs on rare diseases; European Research Council) research funding agencies. Indeed, several of these funding agencies were also acknowledged in the Arisla-derived publications. This indicates how relevant the synergy between international charities supporting research can be, and that seed funding by small foundations triggers novel research, which can then be leveraged through main national and international funding programs.

The information gathered through the PIs' survey was complemented by the analysis conducted by the AriSLA staff on the funded projects. AriSLA grants contributed to the discovery of new genes implicated in ALS onset (Majounie et al., 2012; Wu et al., 2012; Johnson et al., 2014; Pensato et al., 2015; Nicolas et al., 2018). Some PIs have been recipients of several AriSLA FGs, which generated relevant publications, such as those on transactive response DNA-binding protein 43 (TDP-43) proteinopathy (Ayala et al., 2011; Avendaño-Vázquez et al., 2012; Romano et al., 2014; Strah et al., 2020) or autophagy and stress granules (Ganassi et al., 2016; Cristofani et al., 2018; Mediani et al., 2021). Five PIs obtained an FG to continue their research started with a PG, such as those on TDP-43 proteinopathy (Capitini et al., 2014; Vivoli Vega et al., 2019) or FUsed in Sarcoma (FUS) protein and stress granules (Mirra et al., 2017; Rossi et al., 2020). This continuous support was instrumental to move research from basic knowledge to more advanced preclinical steps; see in example studies on alternative splicing of FUS mRNA (Mirra et al., 2017; Rossi et al., 2020), cyclophilin A (Pasetto et al., 2017, 2021), or DNA damage response and TDP-43 (Pessina et al., 2019; Cabrini et al., 2021), which led to new ongoing AriSLA preclinical research projects. At least 3 groups have successfully filed a patent with data obtained with the AriSLA grant. These concerned: (i) the identification of a method for *in vitro* diagnosis of ALS by the detection of exosomal RNAs in cerebrospinal fluid (“METHOD FOR *IN VITRO* DIAGNOSIS OF AMYOTROPHIC LATERAL SCLEROSIS,” WO/2019/116412); (ii) the process for the preparation of a synthetic peptide to be used in the treatment of neurodegenerative diseases and ALS (“PHARMACOLOGICALLY ACTIVE PEPTIDE COMPOUND, PROCESS FOR THE PREPARATION AND USE THEREOF,” WO/2017/158502); and (iii) the identification of an inhibitor of miR-129, relative compounds and pharmaceutical compositions for use in the treatment and/or prevention of ALS and Alzheimer's disease (“INHIBITOR OF MIR-129 AND USES THEREOF,” WO/2020/193709) (Rizzuti et al., 2018; Loffreda et al., 2020).

A preclinical study on energy metabolism contributed to building the rationale (Scaricamazza et al., 2020, 2021) for an ongoing international Phase 2 repurposing clinical trial of the small molecule Trimetazidine (Targeting Metabolic Flexibility in Amyotrophic Lateral Sclerosis (ALS)—Full Text View - ClinicalTrials.gov^14^). AriSLA supported 4 interventional trials to test the efficacy of erythropoietin (Lauria et al., 2015), rapamycin (Mandrioli et al., 2018), the cannabinoid derivative Nabiximols (Riva et al., 2019), or guanabenz (Dalla Bella et al., 2021). Although the results of these studies were not conclusive due to their pilot nature, they added information on the clinical effects of these molecules and contributed to harmonize the Italian clinical ALS network activities. One such study generated knowledge (Dalla Bella et al., 2021) that led to an ongoing, industry-sponsored, international Phase 2 clinical trial to test a new compound with better pharmacodynamic profile for ALS than the one initially studied with the AriSLA trial (Treatment Combining Riluzole and IFB-088 in Bulbar Amyotrophic Lateral Sclerosis (TRIALS Protocol)—Full Text View—ClinicalTrials.gov^14^). AriSLA also supported observational studies on clinical biomarkers related to the involvement of the autonomic nervous system (Cairo et al., 2019) or based on the application of imaging techniques such magnetic resonance (Ferraro et al., 2017; Basaia et al., 2020) or positron emission tomography/computed tomography (Marini et al., 2018; Bauckneht et al., 2020).

A summary of the main scientific outcomes of the AriSLA grant projects is reported in Table 1. More details about the information presented in Figures 1, 2 are available in Supplementary Tables 1, 2.

**Table 1:** Summary of the main scientific outcomes of AriSLA-funded projects.

**Research topics**	**No. funded projects**	**No. projects with published results**	**No. original Articles (2010–2023)**	**Key outcomes**	**Key references**
Autophagy and stress response	13	10	64	Progress from basic to preclinical research	Capitini et al. (2014); Ganassi et al. (2016); Cristofani et al. (2018); Pessina et al. (2019); Vivoli Vega et al. (2019); Cabrini et al. (2021); Mediani et al. (2021)
Genetics and genomics	9	8	48	Contribution to gene discovery	Majounie et al. (2012); Wu et al. (2012); Johnson et al. (2014); Pensato et al. (2015); Nicolas et al. (2018)
Non cell autonomous mechanisms	30	21	51	Progress from basic to preclinical research	Romano et al. (2014); Pasetto et al. (2017, 2021); Strah et al. (2020)
RNA metabolism and epigenetics	25	21	73	Progress from basic to preclinical research IP generation	Ayala et al. (2011); Avendaño-Vázquez et al. (2012); Mirra et al. (2017), Rossi et al. (2020 IP: WO/2019/116412), WO/2020/193709 [also references Rizzuti et al. (2018); Loffreda et al. (2020)]
Excitation and energy metabolism	6	5	17	Preclinical POC (repurposing drug) has led to an international Phase 2 trial (same drug) IP generation	Scaricamazza et al. (2020, 2021), https://clinicaltrials.gov/ct2/show/NCT04788745. IP: WO/2017/158502
Clinical research	15	11	28	4 interventional clinical trials Clinical POC (repurposing drug) has led to an industry-sponsored international Phase 2 trial (new small molecule) Imaging biomarkers	Lauria et al. (2015); Mandrioli et al. (2018); Riva et al. (2019); Dalla Bella et al. (2021), https://clinicaltrials.gov/ct2/show/NCT05508074 Ferraro et al. (2017); Marini et al. (2018); Basaia et al. (2020); Bauckneht et al. (2020)

IP, Intellectual Property; POC, Proof of Concept.

## Discussion

The major goal of disease-oriented funding agencies and charities is to promote cutting-edge research for the development of innovative therapies for their disorders of interest. Although their investment is, in most cases, rather limited to be able to produce a real breakthrough on their own, their clear objectives, rigorous selection criteria established in advance, and their ability to synergy with other stakeholders help make them strong engines of innovation.

Among the organizations operating in the ALS field, many, such as the US ALS Association [Dedicated to Finding a Cure for ALS | The ALS Association (see text footnote 11)], the ALS Canada (https://als.ca/), or the Motor Neuron Disease Association in UK [MND Association | Fighting motor neurone disease (see text footnote 13)] focus their mission both on assistance to patients and research. The evaluation of their portfolio, therefore, includes several areas of impact, ranging from the support to the patient community, advocacy and empowerment to the support to research, including implementation of clinical standards of care, clinical trial facilities and contribution to interventional studies. As such, the broad impact on the patient community is a direct measure of the successful implementation of their strategies and investment, as reported in their annual reports.^15^, ^16^, ^17^ Other organizations are mainly focused on funding research. For instance, Fondation Thierry Latran's objective is to promote ALS research interactions across European countries; according to the information available on their website, the impact of the Foundation is reported as the number of European projects funded at each Call for applications and news on the most relevant publications derived from their grants (see text footnote 12). Like Fondation Latran, AriSLA is fully dedicated to fund research, but its investment is limited at national level. AriSLA considers the regular evaluation of their portfolio of investment a vital practice to monitor the most outstanding achievements and confirm/adjust their strategic directions. However, the notion of “excellence” to evaluate research outcomes remains a rather vague concept (Jong et al., 2021), which is particularly difficult to define, when the impact on patients is not immediate. Bibliometric measures are an important component, but not the only one, and other parameters, such as productivity, quality, reproducibility, sharing of data and resources, and translation, should be considered in a portfolio analysis (Santangelo, 2017). Such integrated approach was adopted by AriSLA to analyze to which extent its funded research was truly ground-breaking and helped move the field toward new therapeutic approaches.

### Assessment of AriSLA-derived research

A first consideration to be made is that AriSLA Foundation has never imposed specific topics in its calls for projects; therefore, the funded projects encompassed several thematic areas, which were/still are hot ALS research topics (Van Damme et al., 2017). Over the years, several groups have received multiple grants, which have helped strengthen a strong Italian research network focused, particularly, on areas such as genetics, autophagy and stress response, and RNA metabolism (Table 1).

These areas have been acknowledged as highly relevant by the Working Groups that concurred to draft the NIH ALS Strategic Planning and the understanding of the molecular mechanisms underlying clinical heterogeneity in ALS is still considered an unmet need^1^. In particular, the role played by TDP-43 (an area well-funded by AriSLA) is considered fundamental to investigate the relationship between familial and sporadic ALS, this protein being a central target to most ALS forms (see text footnote 5 and 6).

At each AriSLA's new call, all applications compete at the same level, with the only criteria for funding decisions based on the scientific quality of the application, which is carefully evaluated by the reviewers. The ex-post evaluation of the results of previously funded projects is one of the driving factors for ex-ante evaluation of research by peers when considering renewal projects. Advances from basic science toward preclinical and translational objectives followed several lines of research, with progression reported in relevant publications derived both from FG and PG projects. Moreover, in the case of PGs, the clear definition of the call's expectations and evaluation criteria contributed also to bringing new experts and younger scientists to the field, who then continued their ALS research activity with the support of new FGs.

In agreement with the DORA declaration principles (see text footnote 10), AriSLA does not assess candidates using journal-based metrics such as the journal impact factor. Therefore, also the analysis of the achievements of its funded research was based on different bibliometric tools, and on an article-level assessment that allows to evaluate to which extent publications derived from its grant projects have been influential in the peer co-citation network. This analysis used the NIH Portfolio Office iCite dashboard, which provides RCR values in comparison with the publications derived from NIH-R01 funded projects for the referring year (Hutchins et al., 2016, 2019a,b; Santangelo, 2017).

AriSLA evaluated the mean and median RCR values of publications acknowledging its grants, highlighting a good performance of these articles against the NIH benchmark. The analysis focused on original articles showed high RCR values in all topics addressed by AriSLA projects, indicating a strong influence on the peer community in all thematic areas (Figure 1) and effective international interactions and leadership of the AriSLA-funded PIs (Figure 2). Interestingly, not only the FGs but also several PGs were very productive, generating publications with high RCR values and stimulating new collaborations, despite their exploratory nature and limited funding.

The ToB provided an intuitive visual snapshot of the type of research funded by AriSLA, with a dual component of human research-related articles (Human vertex of the ToB) and a double-bubble cluster of publications derived from basic and preclinical research at the bottom of the triangle, closer to the Mol/Cell than to the Animal vertex (Figure 3). Given that most of AriSLA projects were in the field of basic research, it is probably still premature to expect a clear transition toward translation. Nevertheless, two interesting trends were observed that according to the developers of the ToB algorithm could be considered as indices of translational potential (Weber, 2013; Hutchins et al., 2019b). First, articles derived from preclinical projects were mapped around the bisector of the Mol/Cell-Animal axis and the Human vertex (although still in the lower part of the triangle). Second, more than 18% of all original articles have already been cited by clinical publications. These articles had very high RCR values and a high number of citations, again indicative of a strong influence on the referring scientific community. Interestingly, the PIs who authored these publications were also those who showed a better performance in networking both at Italian and international levels and were more successful in obtaining multiple funds in hot ALS research areas, both from AriSLA and other national and international agencies.

Recently, the National Amyotrophic Lateral Sclerosis Registry, an academic initiative established by the federal Agency for Toxic Substances and Disease Registry, which is part of the Centers for Disease Control and Prevention, published an analysis of the impact of the Registry-funded ALS research, using the RCR and the ToB for their bibliometric assessment too (Rechtman et al., 2022). Interestingly, in addition to examining the direct results of their funded research, the authors extended the bibliometric analysis to the publications citing their articles, commenting on the impact their Registry- funded projects had in influencing other research to amplify the progress of knowledge.

The greater appropriateness of RCR over the JIF for the analysis of the impact of an article in the community of interest has been proposed by Surkis and Spore (2018). The mean and weighted RCR values (the total RCR scores of the group of articles under consideration) have also been suggested as appropriate tool to compare research productivity in the academic environment in different medical fields (Reddy et al., 2020; Dijanic et al., 2022; Patel and Ali, 2022). The analysis herein presented indicated that the iCite tools could be adopted by (major and minor) funding organizations other than the NIH to evaluate their research investment, by using NIH funding as a reference. Other academic institutions such as the Center for Science and Technology in Leiden, The Netherlands, have developed algorithms for the normalized indicators of citation impact (Šubelj et al., 2016). However, these tools are used only for their institution and are not externally available.

### The clinical impact of AriSLA research

The goal of the Foundation is to promote therapeutic approaches to be validated in clinical setting on people affected by this devastating disease. How to combine this ambitious goal with an investment that is largely in basic and preclinical research? What is considered cutting-edge research in this field and how can a small foundation like AriSLA make a difference? Above all, what significant role can a small funding agency play in such a rapidly changing international scenario?

While it is still too early to see any real impact of AriSLA research on patients, the global assessment presented here has suggested an interesting trend toward translational research, with an ongoing clinical trial that has based its rationale on preclinical proof-of-concept evidence generated through AriSLA grants (Scaricamazza et al., 2020, 2021; Targeting Metabolic Flexibility in Amyotrophic Lateral Sclerosis (ALS)—Full Text View—ClinicalTrials.gov). AriSLA supported 4 clinical trials on drug repurposing (Table 1), of which one provided the proof-of-concept and rationale for an ongoing industry-sponsored trial with a new small molecule expected to cause fewer side effects than the drug tested in the AriSLA-sponsored study (Dalla Bella et al., 2021,^18^). They cannot be considered ground-breaking studies, being “classical” pharmacological clinical trials; nevertheless, if their clinical outcomes are positive, the treatments studied could be valuable and applicable to much of the ALS population, which is a relevant outcome for the patient community.

Interventional clinical trials have become economically unsustainable for a small agency like AriSLA and for this reason since 2018 they have not been admitted to the annual calls for projects. The Foundation still support observational clinical studies and over the years 6 such studies have been funded to explore new clinical biomarkers related to the involvement of the autonomic nervous system (Cairo et al., 2019) or based on the application of imaging techniques such magnetic resonance (Ferraro et al., 2017) or positron emission tomography/computed tomography (Marini et al., 2018; Bauckneht et al., 2020). This type of investment is in line with the suggestions of the NIH Working Groups, which considered the investment in emerging technologies such as clinical imaging involving multidisciplinary collaborations as top priority (see text footnote 5 and 6).

### Limitations of the analysis

The bibliometric analysis performed on AriSLA-derived publications may be biased due to several factors: (i) information available to the AriSLA scientific office could be incomplete, being based only on its funded projects and derived publication; (ii) publications that do not acknowledge AriSLA support may be lost. This more likely applies to review papers than to original articles, given that PIs are required to acknowledge the AriSLA grant, but may perceive the content of a review as less directly related to the project. To minimize loss of publications, the information reported by the PIs in their final scientific reports at the end of their funding period is accurately verified; (iii) the number of publications available for bibliometric analysis was rather limited with respect to the benchmark, particularly for the granular analysis on thematic areas; (iv) the most recently funded projects, which are those more advanced toward preclinical and translational research, did not generate publications yet, or articles were too recent to provide reliable citations and RCR values to be included in the analysis.

A limitation of the RCR-based analysis is that the benchmark is limited to NIH-derived publications. Moreover, RCR values do not take into account the author position within an article, but this analysis was performed by AriSLA‘s staff when deemed important to be dissected out. Citation values derived from Web of Science by Clarivate analytics and the Citation module of iCite by NIH Portfolio Office were based on different citation collections. Despite this, retrieved values were very similar, suggesting the equal value of both platforms for this analysis.

Regarding the information on PIs' performance derived from the AriSLA 2020 survey, it could not be excluded that PIs provided biased responses toward positive rather than negative regarding their success in attracting other funds on their ALS research.

Nevertheless, integration of multiple sources and parameters, including quantitative (benchmark with validated bibliometrics) and qualitative (scientific evaluation, grant monitoring, anecdotal evidence) measures, allowed to track the relevant scientific contribution of several research groups, demonstrating a nice convergence of the information, and limiting the effect of potential biases. This supports the need to match multiple indicators when research performance is evaluated (Santangelo, 2017; Jong et al., 2021).

### General considerations and conclusions

In its 13 years of activity, AriSLA consolidated a strong Italian scientific community and promoted valuable research on ALS whose outcomes greatly contributed to steer the international scientific debate. It could therefore be said that one of the original objectives, which was to promote a cultural growth of research on ALS in Italy, has been achieved. The key elements that laid the foundation for this achievement can be grouped into two main categories. On the one hand, they consisted in a clear definition of the objectives, specifically a call for research with potential impact on patients, no matter how far, and a diversification of the portfolio with *ad hoc* selection criteria. Furthermore, the transparency and rigor of the process and the close interaction of AriSLA stakeholders with scientists, have helped build trust and develop a strong sense of belonging of researchers to AriSLA and its mission. On the other hand, AriSLA has never interfered in the scientific evaluation, fully entrusting the selection decisions to its reviewers. Indeed, the peers' perspective goes beyond their role in grant assessment, being a key factor also in evaluating manuscripts sent to publishers for publication and in their action of citing peer work. Potential biases can impact at any level, and it is the responsibility of funders and editors to have adequate policies to minimize this risk, and an ethical mandate for scientists engaged in all processes of taking a rigorous and consistent approach.

Much remains to be understood at physiopatological level, and innovative treatments can only come to light when the molecular pathways are clear. Therefore, there is still a strong need for original and innovative basic and preclinical research, which needs to be “clinically informed” to contribute knowledge and foster therapeutic developments in ALS (see text footnote 5 and 6). This concept has also been embedded in the new AriSLA strategic plan for the period 2023–2026. The regular monitoring of the most promising results, i.e., those that have already helped ignite the international debate on ALS at clinical level, will be important to better focus its resources and promote clinical translation. Guided by its new strategic plan, AriSLA Foundation will continue to play an important role as a hub for the Italian ALS community, supporting scientists and sharing its research outcomes with patients, who are the main stakeholders and look forward to seeing an improvement in their disease condition.

Proactive interaction with other players in the field is an additional cornerstone of the NIH ALS strategies, which has been adopted by AriSLA. Sharing policies on research funding and assessment among charities with similar objectives can help guide the debate and research directions to be undertaken in specific fields, such as, in the example reported here, that of a rare disease as ALS.

## Data availability statement

The PubMed Identifier dataset used for the analyses reported in the text and in Figures 1–3 is available from the corresponding author on request.

## Author contributions

AA, SG, and MR outlined the structure of the paper and wrote the manuscript. DB and SP (Telethon portfolio office, SP until September 30, 2019) contributed to the bibliometric analysis. TZ contributed to the management of some of the activities described. MM is the President of the Foundation and initiator of AriSLA. All authors read and approved the final manuscript.

## Footnotes

^1^Rare Disease Foundation website: https://rarediseasefoundation.org/ (accessed July 7, 2023).

^2^Juvenile Diabetes Research Foundation website: https://www.jdrf.org (accessed July 7, 2023).

^3^Cystic Fibrosis Foundation website: https://www.cff.org (accessed July 7, 2023).

^4^Parent Project Muscular Dystrophy website: https://www.parentprojectmd.org (accessed July 7, 2023).

^5^National Institutes of Health U.S. - National Institute of Neurological Disorders and Stroke; ‘The ALS strategic planning workshop' website: als-strategic-plan.com (Accessed July 7, 2023).

^6^https://www.ninds.nih.gov/sites/default/files/documents/ALS%20Strategic%20Plan_01_19_23_508C.pdf (Accessed July 7, 2023).

^7^Clarivate Analytics Web of Science Core Collection™ website: www.webofscience.com (Accessed July 7, 2023).

^8^National Institutes of Health U.S. iCite website; https://icite.od.nih.gov/analysis (please use Google Chrome browser (accessed July 7, 2023)).

^9^National Institutes of Health U.S. iCite User Guide, Influence module: https://icite.od.nih.gov/user_guide?page_id=ug_infl (Accessed July 7, 2023).

^10^https://sfdora.org/read/ (Accessed July 7, 2023).

^11^The ALS Society of Canada website: https://als.ca/ (accessed July 7, 2023).

^12^Fondation Thierry Latran, France, website; http://www.fondation-thierry-latran.org/english (Accessed July 7, 2023).

^13^Motor Neuron Disease Association, UK (MNDA), website; https://www.mndassociation.org (Accessed July 7, 2023).

^14^National Institutes of Health U.S. National Library of Medicine ClinicalTrials.gov website: “Treatment Combining Riluzole and IFB-088 in Bulbar Amyotrophic Lateral Sclerosis (TRIALS Protocol)”. https://clinicaltrials.gov/ct2/show/NCT05508074 (accessed July 7, 2023).

^15^Amyotrophic Lateral Sclerosis Association, USA (ALSA) 2021 Annual Report: https://www.als.org/sites/default/files/2022-03/2021-Annual-Report.pdf (Accessed July 7, 2023).

^16^Motor Neuron Disease Association, UK (MNDA), 2021 Annual Report: https://www.mndassociation.org/app/uploads/2022/07/Annual-Report-2021.pdf (Accessed July 7, 2023).

^17^The ALS Society of Canada 2021 Impact Report: https://als.ca/wp-content/uploads/2022/06/ALS-Canada-AR-2021_ENG_FINAL.pdf (accessed July 7, 2023).

^18^National Institutes of Health U.S. National Library of Medicine ClinicalTrials.gov website: “Targeting Metabolic Flexibility in Amyotrophic Lateral Sclerosis (ALS) (MetFlex)”; https://clinicaltrials.gov/ct2/show/NCT04788745 (accessed July 7, 2023).

## References

[B1] Deciphering impact factors. (2003). Nat. Neurosci. 6, 783. 10.1038/nn0803-783

[B2] Aiuti A. Pasinelli F. Naldini L. (2022). Ensuring a future for gene therapy for rare diseases. Nat. Med. 28, 1985–1988. 10.1038/s41591-022-01934-9 35970921

[B3] Aloise F. Aricò P. Schettini F. Riccio A. Salinari S. Mattia D.. (2012). A covert attention P300-based brain-computer interface. Geospell Ergon. 55, 538–51. 10.1080/00140139.2012.661084 22455372

[B4] Avendaño-Vázquez S. E. Dhir A. Bembich S. Buratti E. Proudfoot N. Baralle F. E.. (2012). Autoregulation of TDP-43 mRNA levels involves interplay between transcription, splicing, and alternative polyA site selection. Genes Dev. 26, 1679–1684. 10.1101/gad.194829.112 22855830 PMC3418585

[B5] Ayala Y. M. De Conti L. Avendaño-Vázquez S. E. Dhir A. Romano M. D'Ambrogio A.. (2011). TDP-43 regulates its mRNA levels through a negative feedback loop. EMBO J. 30, 277–88. 10.1038/emboj.2010.310 21131904 PMC3025456

[B6] Basaia S. Agosta A. Cividini C. Trojsi F. Riva N. Spinelli E. G.. (2020). Structural and functional brain connectome in motor neuron diseases: a multicenter MRI study. Neurology. 95, e2552–e2564. 10.1212/WNL.0000000000010731 32913015 PMC7682834

[B7] Bauckneht M. Lai R. Miceli A. Schenone D. Cossu V. Donegani M. I.. (2020). Spinal cord hypermetabolism extends to skeletal muscle in amyotrophic lateral sclerosis: a computational approach to (18F)-fluorodeoxyglucose PET/CT images. EJNMMI Res. 10, 23. 10.1186/s13550-020-0607-5 32201914 PMC7085992

[B8] Belli R. Bonato A. De Angelis L. Mirabilii S. Ricciardi M. R. Tafuri A.. (2019). Metabolic reprogramming promotes myogenesis during aging. Front. Physiol. 10, 897. 10.3389/fphys.2019.00897 31354530 PMC6636331

[B9] Budini M. Buratti E. Stuani C. Guarnaccia C. Romano V. De Conti L.. (2012). Cellular model of TAR DNA-binding protein 43 (TDP-43) aggregation based on its C-terminal Gln/Asn-rich region. J. Biol. Chem. 287, 7512–25. 10.1074/jbc.M111.288720 22235134 PMC3293573

[B10] Cabrini M. Roncador M. Galbiati A. Cipolla L. Maffia A. Iannelli F.. (2021). DROSHA is recruited to DNA damage sites by the MRN complex to promote non-homologous end joining. J. Cell Sci. 134, jcs249706. 10.1242./jcs.249706 33558311 PMC8015226

[B11] Cagan R. (2013). The San Francisco declaration on research assessment. Dis. Model Mech. 6, 869–70. 10.1242/dmm.012955 23690539 PMC3701204

[B12] Cairo B. De Maria B. Bari V. Vaini E. Heusser K. Tank J.. (2019). Information-domain method for the quantification of the complexity of the sympathetic baroreflex regulation in healthy subjects and amyotrophic lateral sclerosis patients. Physiol. Meas. 40, 034004. 10.1088/1361-6579/ab0d4b 30840931

[B13] Caligari M. Godi M. Guglielmetti S. Franchignoni F. Nardone A. (2013). Eye tracking communication devices in amyotrophic lateral sclerosis: impact on disability and quality of life. Amyotroph. Lat. Scler. Frontotemporal. Degener. 14, 546–52. 10.3109/21678421.2013.803576 23834069

[B14] Capitini C. Conti S. Perni M. Guidi F. Cascella R. De Poli. (2014). TDP-43 inclusion bodies formed in bacteria are structurally amorphous, non-amyloid and inherently toxic to neuroblastoma cells. PLoS ONE. 9, e86720. 10.1371/journal.pone.0086720 24497973 PMC3907574

[B15] Cristofani R. Crippa V. Vezzoli G. Rusmini P. Galbiati M. Cicardi M. E.. (2018). The small heat shock protein B8 (HSPB8) efficiently removes aggregating species of dipeptides produced in C9ORF72-related neurodegenerative diseases. Cell Stress Chaperones. 23, 1–12. 10.1007/s12192-017-0806-9 28608264 PMC5741577

[B16] Dalla Bella E. Bersano E. Antonini G. Borghero G. Capasso M. Caponnetto C.. (2021). The unfolded protein response in amyotrophic later sclerosis: results of a phase 2 trial. Brain 144, 2635–2647. 10.1093/brain/awab167 33905493 PMC8557337

[B17] Dijanic N. C. Sudah S. Y. Michel C. R. Smith T. A. Patankar A. Manzi J. E.. (2022). Evaluation of the national institutes of health–supported relative citation ratio among American orthopedic spine surgery faculty: a new bibliometric measure of scientific influence. N. Am. Spine Soc. J. 11, 100143. 10.1016./j.xnsj.2022.100143 35928806 PMC9344340

[B18] Errichelli L. Dini Modigliani S. Laneve P. Colantoni A. Legnini I. Capauto D.. (2017). FUS affects circular RNA expression in murine embryonic stem cell-derived motor neurons. Nat. Commun. 8, 14741. 10.1038/ncomms14741 28358055 PMC5379105

[B19] Ferraro P. M. Agosta F. Riva N. Copetti M. Spinelli E. G. Falzone Y.. (2017). (2017). Multimodal structural MRI in the diagnosis of motor neuron diseases. Neuroimage Clin. 16, 240–247. 10.1016/j.nicl.08002 28794983 PMC5545829

[B20] Gagliardi S. Zucca S. Pandini C. Diamanti L. Bordoni M. Sproviero D.. (2018). Long non-coding and coding RNAs characterization in peripheral blood mononuclear cells and spinal cord from amyotrophic lateral sclerosis patients. Sci Rep. 8, 2378. 10.1038/s41598-018-20679-5 29402919 PMC5799454

[B21] Ganassi M. Mateju D. Bigi I. Mediani L. Poser I. Lee H. O.. (2016). (2016). A surveillance function of the HSPB8-BAG3-HSP70 chaperone complex ensures stress granule integrity and dynamism. Mol Cell 63, 796–810. 10.1016/j.molcel.07021 27570075

[B22] Garam K. Gautier O. Tassoni-Tsuchida E. Ma X. R. Gitler A. D. (2020). (2020). ALS genetics: gains, losses, and implications for future therapies. Neuron. 108, 822–842. 10.1016/j.neuron.08022 32931756 PMC7736125

[B23] Garofalo S. Cocozza G. Porzia A. Inghilleri M. Raspa M. Scavizzi F.. (2020). Natural killer cells modulate motor neuron-immune cell cross talk in models of amyotrophic lateral sclerosis. Nat. Commun. 11, 1773. 10.1038/s41467-020-15644-8 32286313 PMC7156729

[B24] Hirsch J. E. (2005). An index to quantify an individual's scientific research output. Proc. Natl. Acad. Sci. U S A 102, 16569–16572. 10.1073/pnas.0507655102 16275915 PMC1283832

[B25] Hutchins B. I. Baker K. L. Davis M. T. Diwersy M. A. Haque E. Harriman R. M.. (2019a). The NIH open citation collection: a public access, broad coverage resource. PLoS Biol. 17, e3000385. 10.1371/journal.pbio.3000385 31600197 PMC6786512

[B26] Hutchins B. I. Davis M. T. Meseroll R. A. Santangelo G. M. (2019b). Predicting translational progress in biomedical research. PLoS Biol. 17, e3000416. 10.1371/journal.pbio.3000416 31600189 PMC6786525

[B27] Hutchins B. I. Yuan X. Anderson J. M. Santangelo G. M. (2016). Relative Citation Ratio (RCR): a new metric that uses citation rates to measure influence at the article level. PLoS Biol. 14, e1002541. 10.1371/journal.pbio.1002541 27599104 PMC5012559

[B28] Johnson J. O. Pioro E. P. Boehringer A. Chia R. Feit H. Renton A. E.. (2014). Mutations in the Matrin 3 gene cause familial amyotrophic lateral sclerosis. Nat Neurosci. 17, 664–666. 10.1038/nn.3688 24686783 PMC4000579

[B29] Jong L. Franssen T. Pinfield S. (2021). “*Excellence” in the Research Ecosystem: A Literature Review*. (RoRI Working Paper No. 5). Research on Research Institute.

[B30] Kiernan M. C. Vucic S. Talbot K. McDermott C. J. Hardiman O. Shefner J. M.. (2021). Improving clinical trial outcomes in amyotrophic lateral sclerosis. Nat. Rev. Neurol. 17, 104–118. 10.1038/s41582-020-00434-z 33340024 PMC7747476

[B31] Lauria G. Dalla Bella E. Antonini G. Borghero G. Capasso M. Caponnetto C.. (2015). Erythropoietin in amyotrophic lateral sclerosis: a multicentre, randomised, double blind, placebo controlled, phase III study. J. Neurol. Neurosurg. Psychiatry 86, 879–86. 10.1136/jnnp-2014-308996 25595151 PMC4515982

[B32] Loffreda A. Nizzardo M. Arosio A. Ruepp M. D. Calogero R. A. Volinia S.. (2020). miR-129-5p: a key factor and therapeutic target in amyotrophic lateral sclerosis. Prog. Neurobiol. 190, 101803. 10.1016/j.pneurobio.2020.101803 32335272

[B33] Majounie E. Renton A. E. Mok K. Dopper E. G. P. Waite A. Rollinson S.. (2012). Frequency of the C9orf72 hexanucleotide repeat expansion in patients with amyotrophic lateral sclerosis and frontotemporal dementia: a cross-sectional study. Lancet Neurol. 11, 323–30. 10.1016/S1474-4422(12)70043-1 22406228 PMC3322422

[B34] Mandrioli J. D'Amico R. Zucchi E. Gessani A. Fini N. Fasano A.. (2018). Rapamycin treatment for amyotrophic lateral sclerosis: protocol for a phase II randomized, double-blind, placebo-controlled, multicenter, clinical trial (RAP-ALS trial). Medicine. 97, e11119. 10.1097/MD.0000000000011119 29901635 PMC6024184

[B35] Marini C. Morbelli S. Cistaro A. Campi C. Caponnetto C. Bauckneht M.. (2018). Interplay between spinal cord and cerebral cortex metabolism in amyotrophic lateral sclerosis. Brain. 141, 2272–2279. 10.1093/brain/awy152 30730551 PMC6061793

[B36] Masrori P. Van Damme P. Amyotrophic lateral sclerosis: a clinical review. (2020). Eur. J. Neurol. 27, 1918–1929. 10.1111/ene.14393 32526057 PMC7540334

[B37] Mediani L. Antoniani F. Galli V. Vinet J. Carrà A. D. Bigi I.. (2021). Hsp90-mediated regulation of DYRK3 couples stress granule disassembly and growth via mTORC1 signaling. EMBO Rep. 22, e51740. 10.15252/embr.202051740 33738926 PMC8097338

[B38] Miller T. M. Cudkowicz M. E. Genge A. Shaw P. J. Sobue G. Bucelli R. C.. (2022). Trial of antisense oligonucleotide tofersen for SOD1 ALS. N. Engl. J. Med. 387, 1099–1110. 10.1056/NEJMoa2204705 36129998

[B39] Mirra A. Rossi S. Scaricamazza S. Di Salvio M. Salvatori I. Valle C.. (2017). Functional interaction between FUS and SMN underlies SMA-like splicing changes in wild-type hFUS mice. Sci. Rep. 7, 2033. 10.1038/s41598-017-02195-0 28515487 PMC5435706

[B40] Nicolas A. Kenna K. P. Renton A. E. Ticozzi N. Faghri F. Chia R.. (2018). Genome-wide analyses identify KIF5A as a novel ALS gene. Neuron. 97, 1268–1283. 10.1016/j.neuron.02027 29566793 PMC5867896

[B41] Pasetto L. Grassano M. Pozzi S. Luotti S. Sammali E. Migazzi A.. (2021). Defective cyclophilin A induces TDP-43 proteinopathy: implications for amyotrophic lateral sclerosis and frontotemporal dementia. Brain 144, 3710–3726. 10.1093/brain/awab333 34972208 PMC8719849

[B42] Pasetto L. Pozzi S. Castelnovo M. Basso M. Estevez A. G. Fumagalli S.. (2017). Targeting extracellular cyclophilin a reduces neuroinflammation and extends survival in a mouse model of amyotrophic lateral sclerosis. J. Neurosci. 37, 1413–1427. 10.1523/JNEUROSCI.2462-16.2016 28011744 PMC6705677

[B43] Patel P. A. Ali M. J. (2022). The relative citation ratio: a brief primer on the national institutes of health-supported bibliometric. Semin. Ophthalmol. 37, 539–540. 10.1080/08820538.2022.2088981 35695556

[B44] Pensato V. Tiloca C. Corrado L. Bertolin C. Sardone V. Del Bo R.. (2015). TUBA4A gene analysis in sporadic amyotrophic lateral sclerosis: identification of novel mutations. J. Neurol. 262, 1376–8. 10.1007/s00415-015-7739-y 25893256 PMC6614739

[B45] Pessina F. Giavazzi F. Yin Y. Gioia U. Vitelli V. Galbiati A.. (2019). Functional transcription promoters at DNA double-strand breaks mediate RNA-driven phase separation of damage-response factors. Nat. Cell Biol. 21, 1286–1299. 10.1038/s41556-019-0392-4 31570834 PMC6859070

[B46] Rechtman L. Brenner S. Wright M. Ritsick M. Rahman F. Han M.. (2022). Impact of the national amyotrophic lateral sclerosis registry: analysis of registry-funded research. Ann. Clin. Transl. Neurol. 9, 1692–1701. 10.1002/acn3.51660 36259277 PMC9639630

[B47] Reddy V. Gupta A. White M. D. Gupta R. Agarwal P. Prabhu A. V.. (2020). Assessment of the NIH-supported relative citation ratio as a measure of research productivity among 1,687 academic neurological surgeons. J. Neurosurg. 31, 1–8. 10.3171/2019.11.JNS192679 32005024

[B48] Riva N. Mora G. Sorarù G. Lunetta C. Ferraro O. E. Falzone Y. for the CANALS Study Group. (2019). Safety and efficacy of nabiximols on spasticity symptoms in patients with motor neuron disease (CANALS): a multicentre, double-blind, randomised, placebo-controlled, phase 2 trial. Lancet Neurol. 18, 155–164. 10.1016/S1474-4422(18)30406-X 30554828

[B49] Rizzuti M. Filosa G. Melzi V. Calandriello L. Dioni L. Bollati V.. (2018). MicroRNA expression analysis identifies a subset of downregulated miRNAs in ALS motor neuron progenitors. Sci. Rep. 8, 10105. 10.1038/s41598-018-28366-1 29973608 PMC6031650

[B50] Romano G. Klima R. Buratti E. Verstreken P. Baralle F. E. Feiguin F.. (2014). (2014). Chronological requirements of TDP-43 function in synaptic organization and locomotive control. Neurobiol. Dis. 71, 95–109. 10.1016/j.nbd.07007 25088713

[B51] Rossi S. Rompietti V. Antonucci Y. Giovannini D. Scopa C. Scaricamazza S.. (2020). UsnRNP trafficking is regulated by stress granules and compromised by mutant ALS proteins. Neurobiol. Dis. 138, 104792. 10.1016/j.nbd.2020.104792 32027933

[B52] Santangelo G. M. (2017). Article-level assessment of influence and translation in biomedical research. Mol Biol Cell. 28, 1401–8. 10.1091/mbc.E16-01-0037 28559438 PMC5449139

[B53] Scaricamazza S. Salvatori I. Amadio S. Nesci V. Torcinaro A. Giacovazzo G.. (2021). Repurposing of Trimetazidine for amyotrophic lateral sclerosis: a study in SOD1 G93A mice. Br. J. Pharmacol. 179, 1732–1752. 10.1111/bph.15738 34783031 PMC9305494

[B54] Scaricamazza S. Salvatori I. Giacovazzo G. Loeffler J. P. Renè F. Rosina M.. (2020). Skeletal-muscle metabolic reprogramming in ALS-SOD1 G93A mice predates disease onset and is a promising therapeutic target. iScience. 23, 101087. 10.1016/j.isci.2020.101087 32371370 PMC7200935

[B55] Strah N. Romano G. Introna C. Klima R. Marzullo M. Ciapponi L.. (2020). TDP-43 promotes the formation of neuromuscular synapses through the regulation of Disc-large expression in Drosophila skeletal muscles. BMC Biol. 18, 34. 10.1186/s12915-020-00767-7 32216790 PMC7099817

[B56] Šubelj L. van Eck N. J. Waltman L. (2016). Clustering scientific publications based on citation relations: a systematic comparison of different methods. PLoS ONE. 11, e0154404. 10.1371/journal.pone.0154404 27124610 PMC4849655

[B57] Surkis A. Spore S. (2018). The relative citation ratio: what is it and why should medical librarians care? J. Med. Libr. Assoc. 106, 508–513. 10.5195/jmla.2018.499 30271298 PMC6148595

[B58] Van Damme P. Robberecht W. Van Den Bosch L. (2017). Modelling amyotrophic lateral sclerosis: progress and possibilities. Dis. Model Mech. 10, 537–549. 10.1242/dmm.029058 28468939 PMC5451175

[B59] Vasta R. Moglia C. Manera U. Canosa A. Grassano M. Palumbo F.. (2021). What is amyotrophic lateral sclerosis prevalence? Amyotroph Lateral Scler. Frontotem. Degener. 21, 1–6. 10.1080/21678421.2021.1936557 34151660

[B60] Vivoli Vega M. Nigro A. Luti S. Capitini C. Fani G. Gonnelli L.. (2019). Isolation and characterization of soluble human full-length TDP-43 associated with neurodegeneration. FASEB J. 33, 10780–10793. 10.1096/fj.201900474R 31287959

[B61] Weber G. M. (2013). Identifying translational science within the triangle of biomedicine. J Transl Med. 11, 126. 10.1186/1479-5876-11-126 23705970 PMC3666890

[B62] Wu C. H. Fallini C. Ticozzi N. Keagle P. J. Sapp P. C. Piotrowska K.. (2012). Mutations in the profilin 1 gene cause familial amyotrophic lateral sclerosis. Nature. 488, 499–503. 10.1038/nature11280 22801503 PMC3575525

