# Supplementary material for: The positive impact on translational research of Fondazione italiana di ricerca per la Sclerosi Laterale Amiotrofica (AriSLA), a non-profit foundation focused on amyotrophic lateral sclerosis. Convergence of ex-ante evaluation and ex-post outcomes when goals are set upfront
Source: Front Res Metr Anal. 2023 Aug 4;8:1067981. doi: 10.3389/frma.2023.1067981 (PMC10436489; doi:10.3389/frma.2023.1067981)
Supplement: Supplementary file 1 [file Table_1.docx]

|  | **Genetics and genomics** | | | **Autophagy and stress response** | | | **RNA metabolism and epigenetics** | | | **Non cell autonomous mechanisms** | | | **Clinical research** | | | **Excitation and energy metabolism** | | | **All articles** | | |
| --- | --- | --- | --- | --- | --- | --- | --- | --- | --- | --- | --- | --- | --- | --- | --- | --- | --- | --- | --- | --- | --- |
| **Type of Grant** | **Total** | **FG** | **PG** | **Total** | **FG** | **PG** | **Total** | **FG** | **PG** | **Total** | **FG** | **PG** | **Total** | **FG** | **PG** | **Total** | **FG** | **PG** | **Total** | **FG** | **PG** |
| **Mean RCR** | 3.63 | 3.63 | - | 2.20 | 2.30 | 1.51 | 1.81 | 1.95 | 1.34 | 1.89 | 1.67 | 2.25 | 1.61 | 1.60 | 1.66 | 1.48 | 1.60 | 1.42 | 2.17 | 2.30 | 1.69 |
| **Standard error of the mean** | 0.91 | 0.91 | - | 0.30 | 0.34 | 0.26 | 0.25 | 0.31 | 0.24 | 0.24 | 0.20 | 0.53 | 0.20 | 0.22 | 0.61 | 0.27 | 0.59 | 0.30 | 0.19 | 0.23 | 0.21 |
| **Median RCR** | 1.46 | 1.46 | - | 1.58 | 1.58 | 1.88 | 1.32 | 1.37 | 0.97 | 1.60 | 1.53 | 1.69 | 1.48 | 1.49 | 1.05 | 0.97 | 0.92 | 1.02 | 1.49 | 1.50 | 1.31 |
| **No. projects with publications** | 4 | 4 | 0 | 10 | 8 | 2 | 20 | 11 | 9 | 19 | 8 | 11 | 11 | 10 | 1 | 5 | 1 | 4 | 66 | 39 | 27 |
| **No. indexed publications** | 42 | 42 | 0 | 55 | 48 | 7 | 63 | 49 | 14 | 45 | 28 | 17 | 27 | 24 | 3 | 16 | 5 | 11 | 248 | 196 | 52 |

**Table 1_supplementary. Details of the bibliometric analysis conducted on original articles by scientific topic of the referral proposal, related to Figure 1.**

RCR, Relative Citation Ratio; FG, Full Grant; PG, Pilot Grant. Period 2010-2021.
