# Supplementary material for: The positive impact on translational research of Fondazione italiana di ricerca per la Sclerosi Laterale Amiotrofica (AriSLA), a non-profit foundation focused on amyotrophic lateral sclerosis. Convergence of ex-ante evaluation and ex-post outcomes when goals are set upfront
Source: Front Res Metr Anal. 2023 Aug 4;8:1067981. doi: 10.3389/frma.2023.1067981 (PMC10436489; doi:10.3389/frma.2023.1067981)
Supplement: Supplementary file 2 [file Table_2.docx]

|  | **International collaborations** | | | **National collaborations** | | | **Single centre** | | |
| --- | --- | --- | --- | --- | --- | --- | --- | --- | --- |
|  | **Total** | **FG** | **PG** | **Total** | **FG** | **PG** | **Total** | **FG** | **PG** |
| **Mean RCR** | 2.92 | 3.14 | 1.71 | 1.66 | 1.71 | 1.51 | 1.78 | 1.62 | 2.17 |
| **Standard error of the mean** | 0.41 | 0.49 | 0.24 | 0.17 | 0.22 | 0.17 | 0.28 | 0.16 | 0.91 |
| **Median RCR** | 1.77 | 1.88 | 1.53 | 1.13 | 1.18 | 1.13 | 1.24 | 1.40 | 0.99 |
| **No. Projects with pub** | 33 | 24 | 9 | 51 | 33 | 18 | 22 | 13 | 9 |
| **No. indexed publications** | 98 | 83 | 15 | 115 | 88 | 27 | 35 | 25 | 10 |

**Table 2_supplementary materials –Details of the bibliometric analysis conducted on original articles by Author’s collaboration, related to Figure 2.**

RCR, Relative Citation Ratio; FG, Full Grant; PG, Pilot Grant. Period 2010-2021.
